# Single-Session Percutaneous Mechanical Thrombectomy Using the Aspirex^®^S Device Plus Stenting for Acute Iliofemoral Deep Vein Thrombosis: Safety, Efficacy, and Mid-Term Outcomes

**DOI:** 10.3390/diagnostics10080544

**Published:** 2020-07-30

**Authors:** Romaric Loffroy, Nicolas Falvo, Kévin Guillen, Christophe Galland, Xavier Baudot, Emmanuel Demaistre, Léo Fréchier, Frédérik Ledan, Marco Midulla, Olivier Chevallier

**Affiliations:** 1 Department of Radiology, Section of Vascular and Image-Guided Therapy, François-Mitterrand University Hospital, 14 Rue Paul Gaffarel, BP 77908, 21079 Dijon, France; kevin.guillen@chu-dijon.fr (K.G.); marco.midulla@chu-dijon.fr (M.M.); olivier.chevallier@chu-dijon.fr (O.C.); 2 Department of Radiology, Section of Vascular Medicine and Phlebology, François-Mitterrand University Hospital, 14 Rue Paul Gaffarel, BP 77908, 21079 Dijon, France; nicolas.falvo@chu-dijon.fr (N.F.); christophe.galland@chu-dijon.fr (C.G.); x.baudot@wanadoo.fr (X.B.); leo.frechier@chu-dijon.fr (L.F.); frederik.ledan@chu-dijon.fr (F.L.); 3 Department of Biology, Section of Biological Haemostasis and Thrombosis Treatment, François-Mitterrand University Hospital, 14 Rue Paul Gaffarel, BP 77908, 21079 Dijon, France; emmanuel.demaistre@chu-dijon.fr

**Keywords:** deep vein thrombosis, acute iliofemoral thrombosis, endovascular treatment, thrombolysis, percutaneous mechanical thrombectomy

## Abstract

To assess the safety, efficacy and mid-term outcomes of single-session percutaneous mechanical thrombectomy (PMT) for acute symptomatic iliofemoral deep vein thrombosis (DVT) using the Aspirex^®^S device. Retrospective review of 30 patients (women, 23; mean age, 45.5 ± 19.9 years; range, 17–76) who underwent PMT with the 10-French Aspirex^®^S device (Straub Medical AG, Wangs, Switzerland) for acute DVT between December 2015 and March 2019. Procedures were performed by popliteal (*n* = 22) or jugular (*n* = 7) approach, or both (*n* = 1). Mean time from diagnosis to PMT was 5.5 ± 4.6 days (range, 2–11). Successful thrombus removal and venous patency restoration were achieved in all patients (100%). Fluid removal was 307.8 ± 66.1 mL (range, 190–410). Additional venous stenting rate was 100%. Mean procedural time was 107.3 ± 33.9 min (range, 70–180). No major complication occurred. The patient’s postprocedural course was uneventful in all cases, with hospital discharge within 2 days in 83.3%. Early in-stent rethrombosis occurred within 1 week in 3 patients, successfully managed by endovascular approach. Secondary stent patency rate was 86.7% at a mean follow-up of 22.3 ± 14.2 months (range, 6–48), as assessed by Duplex ultrasound. Single-session of PMT using the Aspirex^®^S device is a safe and effective therapeutic option in patients presenting with acute symptomatic iliofemoral DVT.

## 1. Introduction

Deep vein thrombosis (DVT) is a significant health care and socio-economical problem, with an incidence of 1 per 1000 per year in Caucasian population [1]. Despite universal usage of anticoagulants, post-thrombotic syndrome (PTS) is the most frequent long-term complication of lower limb DVT, affecting about 23% to 60% of patients within 2 years even with an adequate treatment, major symptoms being chronic pain and edema of the lower limb [1,2]. PTS is severe in 5–10% of cases degrading the quality of life and inducing an economic burden [3,4]. Proximal thrombosis, involving iliac veins, is more at risk of PTS. The frequency of this complication led to its definition as clinical entity by the European Venous Forum [5]. So far, there is no specific medical treatment for PTS. The outcomes of surgical procedures remain poor, leading to the first venous angioplasties in the late 90s in case of chronic obstruction or PTS. Since then, percutaneous endovascular venous recanalization techniques have shown encouraging results in terms of safety and effectiveness in the literature [6,7,8,9,10,11,12]. The Aspirex^®^S device allows pure mechanical rotational thrombectomy and consists of a rotating over-the-wire device designed for efficient and fast removal of occluding material. This study aimed to assess the safety, efficacy, and mid-term outcomes of single-session percutaneous mechanical thrombectomy (PMT) for acute symptomatic iliofemoral deep vein thrombosis (DVT) using the Aspirex^®^S device.

## 2. Materials and Methods

### 2.1. Study Population

From December 2015 to July 2019, all patients with acute (<14 days since symptom onset) symptomatic ascending or descending ilio-femoral deep vein thrombosis (DVT) treated with PMT using the Aspirex^®^S device were included in our single-center observational cohort. Occluded venous segment was evaluated according to the lower extremity thrombosis (LET) classification [13]. Patients with acute symptomatic thrombosis, which were LET class II–III, III, III–IV ones, were referred to our Interventional Radiology Department for PMT. Non-thrombotic obstructive condition, chronic thrombosis, isolated distal LET I and LET II DVT, and asymptomatic patients were exclusion criteria. There was no inclusion criteria restriction pending multidisciplinary decision. This study was performed in compliance with the requirements of the institutional review board.Due to the retrospective nature of the study, our Ethics Committee waived the requirement for informed patient consent.

### 2.2. Imaging Evaluation

Venous duplex ultrasound (DUS) was used prior to treatment to diagnose and localize the DVT. Patients had a DUS (Thoshiba Aplio i800, Canon Medical Systems, Tustin, CA, USA) with a 5 MHz convex array transducer from the inferior vena cava (IVC) to the groin and with a high frequency linear array transducer (8 MHz) from the groin to the foot of both legs before the procedure, at 1 day, 1, 3, 6, 12 months after the procedure, and then annually. Iliac, femoral, popliteal, deep calf veins and vena cava were evaluated in the longitudinal and axial planes. Mid-term PTS was diagnosed by the angiologist on the basis of clinical and DUS features. Every patient had a computed tomography angiography (CTA) scan (Somatom^®^ Definition Flash, Siemens AG, Erlangen, Germany) before the procedure to make an accurate assessment of topography and extension of thrombosis and to evaluate presence of pulmonary embolism. CTA scan at day 1 post-procedure was also systematically performed to evaluate the stents’ integrity, patency and morphology, and the presence of pulmonary embolism. Protocol was standardized: patients received iodinated contrast agent 400 mg/mL (Iomeron 400, Bracco, Milano, Italy) through the forearm (0.6 gI/kg at a rate of 4 mL/s), and the acquisition ranged from the neck to the diaphragm at 30 s after intravenous injection and from the diaphragm to the knees at 100 s after intravenous injection. Reading and interpretation in frontal, axial and sagittal planes and three-dimensional (3D) reconstructions were carried out.

### 2.3. Villalta Score Assessment

Severity of PTS was evaluated at mean follow-up post-procedure for each patient by the Villalta scale being relevant in this pathology according to the literature, except at the acute phase [14]. This scale is based on clinical examination of both legs, including five symptoms (heaviness, pain, cramps, pruritus, and paresthesia) and six signs (edema, induration, hyperpigmentation, new venous ecstasies as varicose veins, redness, and pain during calf compression) scored from 0 to 3. A total score of 0–4 = no PTS, 5–9 = mild PTS, 10–14 = moderate PTS, and 15 or more = severe PTS. Pain and edema were also analyzed separately. Last follow-up was taken into account for evaluation of PTS for each patient, ranging from 6 to 48 months.

### 2.4. Mechanical Thrombectomy Procedure

All procedures were performed by two interventional radiologists (RL and MM, with 15 and 10 years of experience in endovascular therapeutics, respectively) using a Philips Allura Clarity Xper FD20 angio room (Philips, Amsterdam, The Netherlands). The procedure was always performed under conscious sedation and local anesthesia, using 5 to 10 mL of lidocaine hydrochloride 20 mg/mL. All patients received 5000 IU of heparin sodium intravenously at the beginning of the procedure and every 45 min. Then, contralateral transjugular or ipsilateral popliteal cannulation or both permitted a selective venography of the cavo-ilio-femoral veins through a 45 cm length 10-Fr Cook Flexor (Cook Europe, Bjaeverskov, Denmark) sheath. Venous puncture and catheterization were always carried out under DUS control. A retrievable ALN filter (ALN, Bormes les Mimosas, France) was placed into the IVC when needed at the investigator’s discretion either by transjugular or contralateral popliteal approach in prone position, and removed between 1 and 2 months later. Indications for IVC filter implantation were: all LET IV DVT patients and extensive LET III DVT patients at the discretion of the operator. After venography, ilio-femoral thrombosis was crossed using a 0.035-inch Terumo stiff hydrophilic guidewire (Terumo Interventional Systems, Tokyo, Japan). Then, a 4-Fr catheter (Vertebral catheter, Cook Europe, Bjaeverskov, Denmark) was used and the 0.025-inch guidewire of the Straub Medical set was placed into the vessel. Thrombectomy was performed with a 10-Fr 110 cm Aspirex^®^S catheter over the guidewire in all patients. Aspiration was then achieved from popliteal vein to proximally until restoration of the flow. Patency of venous system was tested with control venography after the procedure. In a second time, more or less extensive stenting was performed in all patients to treat the underlying stenosis, mainly at the proximal level. Stents were placed from the IVC or common iliac vein to a landing zone below in a stenosis-free location. Only dedicated self-expandable nitinol venous stents were deployed (Nickel Titanium alloy, Protege GPS Self-Expanding Peripheral System, Covidien, Plymouth, MA, USA; or Sinus-XL Flex Stent or Sinus Superflex-635, Optimed, Ettlingen, Germany) with high radial force to remain stable in the vein due to axial and longitudinal flexibility. Diameter of stents ranged from 12 (common femoral vein) to 16 mm (common iliac vein). A final angioplasty was achieved to restore a good stent caliber. A final venogram in 2 orthogonal planes ensured the final positioning and stent patency. The sheath was removed and pressure was applied for 5 to 10 min at puncture site. Most of the patients were hospitalized overnight and returned to their normal daily activities after 48 h. In some patients, ambulatory procedure was performed with discharge the same day. Heavy physical activities were contraindicated during 15 days. Class II compression socks were recommended for all patients. In the absence of consensus, each patient received 3 months of dual-therapy: low-molecular weight heparin for 1 month transitioned to novel oral anticoagulant for 2 months and antiplatelet drug (acetylsalicylic acid per os 100 mg per day) for 3 months. Anticoagulation was continued based on international guidelines for treatment of DVT and/or pulmonary embolism, according to the presence or not of underlying thrombogenic condition.

### 2.5. Data Collection

In the framework of this study, we retrospectively collected each patient’s demographic data, clinical presentation, underlying etiology and interventional data. Demographic data included age and gender, whereas interventional data included endovascular treatment, the nature of the procedure, the culprit treated vessel, the technical and clinical success, the patency of the native vein, the clinical and DUS features at 1 day, 1 week, 1 month, 6 months and mean follow-up, and the complications and recurrence following the procedure. Technical success was defined as restoration of proximal ilio-femoral blood flow and residual thrombus <20% with patency of the targeted vein on final angiography. Immediate clinical success was defined as relief of acute symptoms within 1 week. Complications were recorded and classified as minor or major complications according to the Society of Interventional Radiology classification system [15]. Patency was defined as target lesion restenosis <50% in DUS after the initial procedure (primary patency rate) or after revision (secondary patency rate). Severity of PTS was assessed using Villalta score measured at mean follow-up.

### 2.6. Statistical Analysis

Descriptive statistics and parameters, such as frequencies and percentages, were used and provided in order to accurately describe our experience regarding the PMT procedure with the Aspirex^®^S device. Values were presented as means ± SDs for variables with normal distribution. Kaplan–Meier curves regarding primary and secondary patency were provided.

## 3. Results

### 3.1. Patient Characteristics

In total, 30 patients (23 women, 7 men; mean age, 45.5 years; range, 17–76) with acute (<14 days since symptom onset) symptomatic ascending or descending ilio-femoral DVT treated with PMT using the Aspirex^®^S device were included in the study. Baseline patients’ characteristics are summarized in Table 1. Occlusion was considered acute in all patients (100%). The left side was involved in most cases (83.3%). According to the LET classification, venous involvement was up to the common iliac vein in 25 (83.3%) of the 30 patients (LET III DVT, meaning involvement of the common femoral vein, external iliac vein, and common iliac vein), with additional involvement of the femoral vein and popliteal vein in 6 of the 25 patients, and up to the inferior vena cava in 5 (16.7%) of the 30 patients (LET IV DVT, meaning LET III DVT with additional involvement of the IVC). None of the last 5 patients had involvement of the femoral vein. In 66.7% of the patients, May–Thurner syndrome represented the underlying etiology, in 30% the etiology remained undetermined. All patients had acute symptoms consisting of swelling and pain. Three (10%) of the 30 patients had additional pulmonary embolism at diagnosis.

### 3.2. Safety and Procedural Outcomes

**Figure 1:**
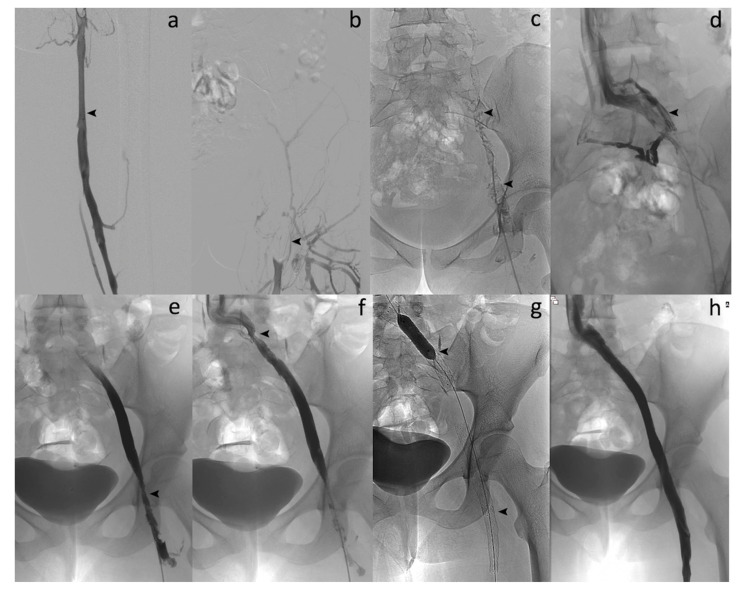
Acute symptomatic ilio-femoral LET III DVT in a 35-year-old woman. (**a**) Left lower extremity venogram in prone position by popliteal approach shows patent femoral vein (arrow). (**b**) Venography reveals no flow within the left common femoral vein (arrow). (**c**) Phlebography confirms complete thrombosis of the left iliofemoral vein (arrows). (**d**) Enlargement of the thrombosed common left iliac vein segment (arrow). (**e**) Venography after percutaneous mechanical thrombectomy (PMT) with the 10-French Aspirex^®^S device shows flow restoration but narrowing at the common femoral vein level (arrow). (**f**) Opacification shows outflow obstruction at the common iliac vein level, too (arrow). (**g**) Stent placement at both venous levels with balloon dilation (arrows). (**h**) Final venography after stent placement reveals successful recanalized common iliac/femoral flow with disappearance of symptoms within 2 days and no recurrence.

The popliteal approach was preferred in most of patients (73.3%) (Figure 1). A temporary retrieval ALN IVC filter was used in 11 (36.7%) of the 30 patients and removed between 1 and 2 months after the procedure. The technical success rate was 100%. No lytic therapy infusion through any catheter left in place was needed. The stenting rate was 100%. No major complications, such as bleeding or additional pulmonary embolism, occurred. No blood transfusion was needed. Two minor complications were reported: a broken and lost wire that was snared in 1 patient; and a broken device helix outside the patient. No device-related complications occurred. Treatment duration ranged from 70 to 180 min. In 5 of the cases, a 10 mg tissue-plasminogen activator (t-PA) bolus injection was performed into the occluded venous segment to complete the procedure, only as an adjunctive medication, at the discretion of the interventional radiologist. No catheter-directed thrombolysis (CDT) with catheter infusion was needed. A mean of 2.3 dedicated venous stents were implanted per patient. Procedural details and safety data are given in Table 2 and Table 3.

### 3.3. Patency and Clinical Success

**Figure 2:**
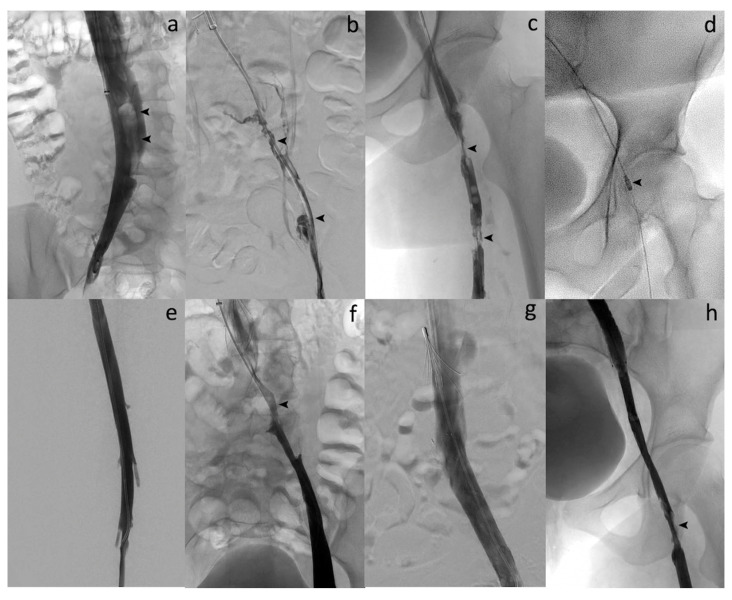
Acute symptomatic ilio-femoral LET IV DVT in a 29-year-old woman. (**a**) Cavography in supine position through ALN IVC filter by jugular approach shows flotting thrombus in the IVC (arrows). (**b**) Venography after catheterization reveals complete thrombosis of the left iliofemoral vein (arrows). (**c**) Phlebography demonstrates partial thrombosis of the left proximal femoral vein too (arrows). (**d**) Over-the-guidewire PMT aspiration with the 10-French Aspirex^®^S device (arrow). (**e**) Venography after PMT shows complete thrombus removal and flow restoration within the left femoral vein. (**f**) Phlebography after aspiration reveals underlying stenosis of the left common iliac vein with May–Thurner syndrome suspicion (arrow). (**g**) Final venography after large stent deployment at the common iliac level shows complete restoration of the flow and successful recanalization of the vein. (**h**) Final phlebography demonstrates residual thrombus attached to the wall of the common femoral vein without significant stenosis leading to no additional stenting. The procedure was technically successful as defined in our study, and further anticoagulation therapy led to complete patency of the common femoral vein at follow-up.

**Figure 3:**
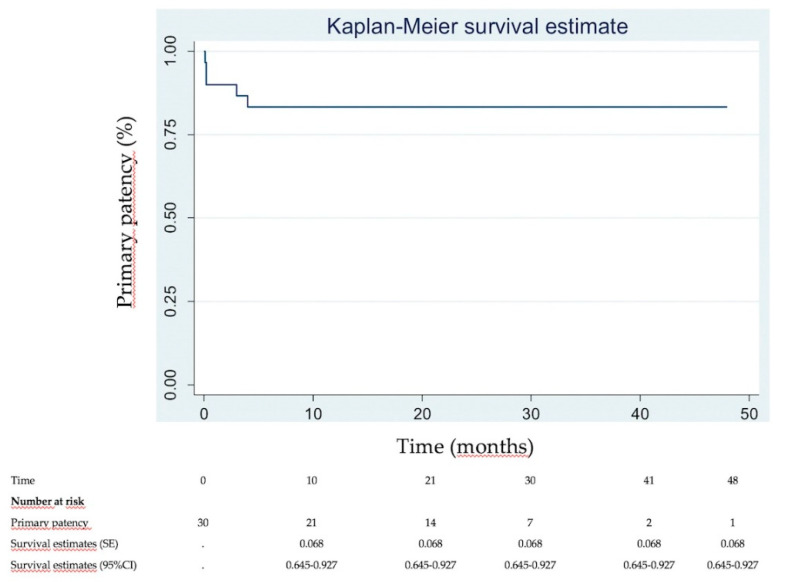
Kaplan–Meier primary patency analysis.

**Figure 4:**
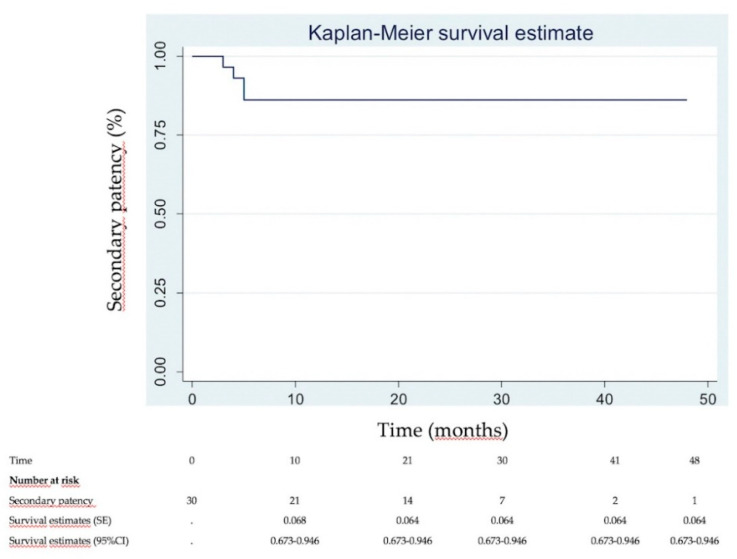
Kaplan–Meier secondary patency analysis.

Immediate clinical success was 90% (Figure 2). None of the patients needed intensive care unit (ICU) stay. Most of patients were discharged within 2 days (83.3%). DUS revealed venous patency in 27 (90%) of the 30 patients at 1 week. Three patients had early in-stent re-thrombosis within 1 week, all successfully managed with new thrombectomy using the Aspirex^®^S device again. Four patients had delayed in-stent re-thrombosis. Among them, 2 already had early in-stent re-thrombosis among the 3 initial patients managed for that. Overall, the primary patency rate was then 83.3% (25/30). Among the 4 patients with delayed in-stent re-thrombosis, recanalization failed in 2 patients, and no recanalization was attempted in the other 2 patients because they were not very symptomatic. Secondary patency rate at mean follow-up of 22.3 ± 14.2 months (range, 6–48) was then 86.7% (26/30 patients). Figure 3 and Figure 4 show Kaplan–Meier curves regarding primary and secondary patency rates, respectively. At mean follow-up, PTS analysis could reveal low PTS symptoms reflected by a Villalta score between 5 and 9 in 73.3% of the patients. Moderate PTS symptoms reflected by a Villalta score between 10 and 14 was seen in 26.7%. No severe PTS (Villalta score ≥ 15) was observed. Overall, mean Villalta score in the “low Villalta score category” (*n* = 22) was 6.8. Mean Villalta score in the “moderate Villalta score category” (*n* = 8) was 11.7. No Villalta score ≥ 15 was noted. One patient died during follow-up from cancer. The outcomes are shown in Table 3.

## 4. Discussion

The present study evaluated the safety, efficacy and mid-term outcomes of single-session PMT for acute symptomatic iliofemoral DVT using the Aspirex^®^S device. We showed that the system, in combination with placement of dedicated venous stents for co-treatment of the underlying lesion, provided excellent technical success (100%), primary patency (90%), and secondary patency (86.7%) rates at a mean follow-up of 22.3 months. No major adverse events occurred. Severe PTS could be prevented in all patients at mean follow-up.

According to the guidelines from the American College of Chest Physicians (ACCP), the anticoagulation therapy is the gold standard treatment modality for the treatment of DVT, and it can help prevent recurrence of venous thromboembolism [16]. However, using anticoagulation therapy alone, 25% to 50% of DVT patients are at risk of developing PTS because of the lack of thrombolytic activity [1,3,16]. Indeed, venous thrombi in the legs are often large and associated with complete venous occlusion. In these cases, thrombolytic agents act on the surface of the clot but may not be able to penetrate and lyse the entire thrombus. Currently, ACCP consensus guidelines recommend thrombolysis when there is a low risk of bleeding [16]. Efforts to prevent the development of PTS have led to the use of advanced endovenous techniques, such as catheter-directed thrombolysis (CDT) and PMT, over the last decade [6,7,8,9,10,11,12]. Until now, CDT was recommended for the treatment of acute proximal DVT of the leg by the guidelines of ACCP in selected cases [16]. However, to date, endovascular treatment options for the management of acute iliofemoral DVT remain controversial because of inconclusive study results [16,17,18,19,20]. Indeed, several studies reported results of CDT and PMT in the literature [6,7,8,9,10,11,12,16,17,18,19,20]. The randomized controlled CaVenT trial included 189 patients with acute iliofemoral DVT divided into 2 groups as anticoagulant therapy alone and anticoagulant therapy plus CDT [19]. A statistically significant decrease in PTS within 2 years was shown in the CDT group compared to the PMT group. These data were confirmed in a meta-analysis demonstrating that additional CDT seemed to be more effective than anticoagulants alone in improving the venous patency and preventing PTS [21].

More recently, data from the randomized controlled ATTRACT trial raised concerns about the benefit of endovascular procedures in comparison with standard-of-care anticoagulant therapy [20]. The main devices used in the trial included the AngioJet rheolytic thrombectomy system, the Trellis peripheral infusion device and catheter-directed rt-PA infusion via a multi-side hole infusion catheter. In ATTRACT, percutaneous CDT failed to significantly decrease the occurrence of PTS when compared to its occurrence in patients treated with anticoagulation alone. At 2-years follow-up, PTS occurred in 46.7% of patients with percutaneous CDT versus 48.2% with anticoagulation alone, whereas bleeding complications were significantly increased. The ATTRACT study had several limitations which can explain the discrepancy with our excellent results with PMT. First, we used the Aspirex^®^S device which presents the advantage of being a pure rotational PMT system avoiding the need for lytic infusion and subsequently decreasing the risk of bleeding complications. None was reported in our series. Second, patients were highly selected in our study and only patients with proximal iliofemoral DVT, meaning with high risk for developing PTS, were treated with PMT. Acute LET I and LET II DVT involving only distal veins below the common femoral vein was an exclusion criteria for treatment. It could have led to prevent severe PTS better than in the ATTRACT study where even patients with distal DVT were included. Third, it has been shown that 80% of patients with acute left iliofemoral DVT present underlying cause of outflow obstruction, such as May–Thurner syndrome, necessitating endovascular treatment [22,23,24,25]. The stenting rate in our cohort was 100% after flow restoration in order to ensure vein patency, while the rate of stenting in the ATTRACT trial was only 28%. Our study on thrombectomy using the Aspirex^®^S device has shown promising results to prevent severe PTS in mid-term and improve the patency of the iliofemoral venous system, without safety concerns in the endovascular treatment of acute iliofemoral DVT and without adjunctive thrombolysis. Interestingly, in our cohort, despite failure or no attempt of recanalization after in-stent re-thrombosis, none of the 4 patients had severe PTS on the mid-term, meaning that PMT at the acute phase does not worsen the symptoms in case of in-stent re-thrombosis. Information about the outcomes of the Aspirex^®^S device is based on data from only two recently published observational studies [26,27]. There are currently no randomized, controlled trial studying outcomes for this system in the literature. Our outcomes were comparable to the outcomes of these published studies. However, in the study by Ozpak et al., balloon angioplasty with or without stent implantation was performed in only three of 21 patients with stenosis in femoral or iliac veins, whereas venous patency was restored in 18 (85%) of the 21 patients at 6 months [26]. The authors concluded that PMT with Aspirex^®^S device can be used alone in the treatment of acute DVT of lower extremities. For the same reasons, such as described in the ATTRACT study, this is not our experience, and we recommend stent placement as an adjunctive therapy in most of patients. The present study also demonstrated that the Aspirex^®^S rotational thrombectomy device was fast, safe and effective. Moreover, our study has the longest mean follow-up time of 22.3 months.

Prophylactic placement of a temporary IVC filter prior to PMT remains controversial. Some authors advocated the use of selective prophylactic IVC filter placement before the PMT procedure, whereas some others recommended its routine use for all patients undergoing PMT [28,29]. In our study, we systematically placed a retrievable IVC filter in LET IV DVT patients in order to protect them from possible pulmonary embolism. Overall, an IVC filter was placed in 36.7% of patients at the operator’s discretion. No pulmonary embolism was observed. We think that selective prophylactic IVC filter placement may be the most appropriate approach instead of routine IVC filter placement. In all cases, the IVC filter should not be removed during the same session because of the risk of thrombus embolization into the retrievable filter, but not too late because of the potential difficulties in explanting it.

The present study had several limitations. First, the most important limitation of the study was the retrospective nature and relatively small sample size. Second, there was no control group and no long-term outcomes of the study population. Our study was limited to only a 22.3-month follow-up with a PTS evaluation available only at last follow-up for each patient. However, to the best of our knowledge, with a mean follow-up of 22.3 months, this study reports the longest mean follow-up time after PMT procedure using the Aspirex^®^S rotational thrombectomy device in the literature. Thus, we think that the mid-term outcomes of the Aspirex^®^S device show its efficiency in the prevention of PTS following acute iliofemoral DVT. Long-term studies with PMT, as well as prospective, controlled trials comparing PMT devices, are needed to confirm results of the present study.

## 5. Conclusions

In conclusion, early removal of thrombus burden with PMT using the Aspirex^®^S catheter is a safe, fast, and effective treatment modality in the management of acute DVT of lower extremities in selected cases in a single session, avoiding the need for CDT. Severe PTS could be prevented in all patients at mid-term follow-up. The device, in combination with placement of dedicated venous stents for co-treatment of the underlying lesion, provided excellent primary and secondary patency rates at mean follow-up. Further studies with larger cohorts are warranted to document the actual safety and confirm long-term outcomes of this endovascular procedure.

## Figures and Tables

**Table 1:** Baseline characteristics of the 30 enrolled DVT patients.

Variable	N (%)
Age, years	45.5 ± 19.9 (17–76)
Men	7 (23.3)
Women	23 (76.7)
DVT history	15 (50)
Known thrombophilic abnormality	6 (20)
Coexistent malignancy	4 (13.3)
Previous IVC surgical ligation	1 (3.3)
On-going anticoagulant therapy	8 (26.7)
On-going antiplatelet medication	4 (13.3)
DVT side involved (left/right/both)	25 (83.3))/4 (13.3)/1 (3.3)
DVT location (LET III/LET IV)	25 (83.3)/5 (16.7)
Etiology (May–Thurner/IVC surgery/other)	20(66.7)/1 (3.3)/9 (30)
Duration of symptoms, days	5.5 ± 4.6 (2–11)
Symptoms (swelling/pain/additional PE)	30 (100)/30 (100)/3 (10)

DVT, deep vein thrombosis; IVC, inferior vena cava; LET, lower extremity thrombosis [13]; PE, pulmonary embolism. Data are expressed as mean ± SD (range) or N (%).

**Table 2:** Procedural details and immediate results.

Variable	N (%)
Approach (jugular/popliteal/both)	7 (23.3)/22 (73.3)/1 (3.3)
Retrieval IVC filter use	11 (36.7)
PMT technical success	30 (100)
CDT with catheter infusion/10 mg t-PA IV bolus injection)	0 (0)/5 (16.7)
Stenting rate	30 (100)
No. of implanted stents	2.3 ± 1.6 (1–4)
No. of Aspirex runs	2.6 ± 1.2 (2–4)
Amount of blood/thrombus aspirated, mL	307.8 ± 66.1 (190–410)
Aspirex PMT run time, min	4.9 ± 0.99 (3.2–6.7)
Total procedural time, min	107.3 ± 33.9 (70–180)
Scopy time, min	20.2 ± 7.7 (8–44)

IVC, inferior vena cava; PMT, percutaneous mechanical thrombectomy; CDT, catheter-directed thrombolysis; t-PA, tissue-plasminogen activator; IV, intravenous; No., number. Data are expressed as mean ± SD (range) or N (%).

**Table 3:** Safety, efficacy, and mid-term outcomes.

Variable	N (%)
Immediate clinical success	27 (90)
Complications (minor/major)	2 (6.7)/0 (0)
Device malfunction	1 (3.3)
Overall hospital stay, days	2.6 ± 2.1 (1–6)
ICU stay, days	0 (0)
Discharge ≤ 2 days	25 (83.3)
Follow-up, months	22.3 ± 14.2 (6–48)
Overall survival	29 (96.7)
Procedure-related mortality	0 (0)
Primary patency rate	25 (83.3)
Secondary patency rate	26 (86.7)
PTS at mean follow-up (low/moderate/severe)	22 (73.3)/8 (26.7)/0 (0)

ICU, intensive care unit; PTS, post-thrombotic syndrome. Data are expressed as mean ± SD (range) or N (%).
